# Valve-Sparing Aortic Root Reimplantation: Early- and Mid-Term Outcomes

**DOI:** 10.1055/a-2642-8919

**Published:** 2025-07-19

**Authors:** Joshua R. Chen, Vishal N. Shah, Scott H. Koeneman, Colin King, Jacqueline McGee, Konstadinos Plestis

**Affiliations:** 1 Division of Cardiac Surgery, Thomas Jefferson University Hospital, Philadelphia, Pennsylvania; 2 Division of Biostatistics and Bioinformatics, Thomas Jefferson University, Philadelphia, Pennsylvania

**Keywords:** valve-sparing root reimplantation, David procedure, root aneurysm, upper, hemisternotomy

## Abstract

**Background:**

Valve-sparing root replacement (VSRR) is an alternative to traditional valve-replacing root replacement. We examined early- and mid-term outcomes after VSRR.

**Methods:**

We performed a retrospective review of a prospectively maintained aortic registry. All patients undergoing VSRR from 2005 to 2023 were included. Statistical analysis was performed in R version 4.3.1. Kaplan–Meier curves were used to describe mortality and freedom from mortality, aortic insufficiency (AI) > 1 + , and aortic valve-related reoperation.

**Results:**

Eighty-one patients underwent VSRR, 59 (72.8%) through full sternotomy (FS) and 22 (27.2%) through upper hemisternotomy. There were no cases of AI > 1+ in the perioperative period, 1 (1.2%) stroke, and no in-hospital mortality. Mean intensive care unit and hospital stay were 3 and 7 days, respectively. Mean follow-up time was 8 years. Freedom from all-cause mortality at 1, 5, and 10 years was 100, 96.6, and 94.4%, respectively. Composite freedom from reoperation, recurrence, or mortality at 1, 5, and 10 years was 98.8, 92.1, and 87.3%, respectively.

**Conclusion:**

With careful preoperative selection, VSRR is a durable procedure for patients with aortic root aneurysm.

Traditionally, root aneurysms have been treated with valve-replacing root replacement (VRRR) procedures, in which the aortic valve and root are replaced with a composite valve–graft conduit. 1 Valve-sparing root replacement (VSRR) procedures, first described by David and colleagues with the root reimplantation technique, are safe alternatives to VRRR procedures in carefully selected patients with normal or repairable cusp morphology. 2 3 4 5 Unlike VRRR procedures, VSRR procedures allow for the avoidance of anticoagulation and theoretically have decreased risk of thromboembolic events often associated with mechanical valves and decreased risk of structural valve degeneration often associated with bioprosthetic valves. 4 5 6

Modifications to the original reimplantation technique have improved valve dynamics and allowed for a more natural restoration of the structure and function of the aortic root while also increasing reproducibility. 7 8 9 10 11 Minimally invasive adaptations to perform VSRR through an upper hemisternotomy (UHS) have also been reported, with good midterm outcomes. 12 13 14 15 In this paper, we aim to share our experience and mid-term outcomes with VSRR for diverse indications using the reimplantation technique.

## Methods

### Study Design

Between August 2005 and January 2023, 84 patients underwent VSRR procedures by a single surgeon (K.A.P.). Of these 84 patients, 3 patients who utilized the remodeling technique were excluded from analysis. All patients, including those undergoing reoperations with a history of prior cardiac or aortic surgery, those with concomitant procedures, and those requiring emergency repair of acute type A aortic dissections were included in the study. Mid-term outcomes include freedom from mortality, freedom from recurrence of aortic insufficiency (AI) (greater than mild), and freedom from aortic valve-related reoperation.

Patients were captured from a prospectively compiled Aortic Wellness Database containing preoperative, intraoperative, and postoperative data, and their data were analyzed, retrospectively. Institutional review board approval for use of the deidentified database for analysis (number: 20D.802) and the study (iRISID-2023-1860) was obtained on January 31, 2023. Informed consent was waived due to the retrospective nature of the study.

### Selection

Patients with aortic root aneurysms (>5.0 cm in diameter) and normal or repairable (small fenestrations, lack of calcifications, pliable leaflets with or without prolapse) cusp morphology with or without AI were considered for surgery. For patients with connective tissue disorders, careful case-specific considerations are made between the patient and a multidisciplinary aortic team to operate at lower thresholds.

### Preoperative Evaluation

Echocardiography is used to assess valve anatomy (bicuspid aortic valve (BAV) vs. tricuspid (TAV)), severity and mechanism of AI, size of the aortic annulus, presence of cusp calcification, stenosis, prolapse, perforation, and ventricular size and function. Any degree of stenosis or cusp calcification impairing leaflet pliability noted on echocardiography are contraindications to VSRR procedures. Contrast-enhanced computed tomography scan with 3-dimensional reconstruction is used to assess root size, morphology, and the need for concomitant aortic procedures. Preoperative pulmonary function testing and coronary angiography are performed in all patients.

### Surgical Technique

**Video 1** How we perform valve-sparing root reimplantation.


We have previously written in great detail about our technique ( Video 1 , available in the online version). 14 In short, median sternotomy is performed and after cannulation, cardiopulmonary bypass (CPB) is initiated. The aorta is cross-clamped and the heart is arrested with cold cardioplegia. The aorta is transected 1 cm above the sinotubular junction (STJ) and three commissural sutures are placed. The aortic annulus, cusps, and sinuses of Valsalva are inspected. The leaflets are evaluated for pliability, prolapse, fenestrations, and calcifications. Any degree of calcification and multiple, large fenestrations are contraindications to VSRR procedures.

If prolapse is identified, it is repaired using free-edge plication prior to valve reimplantation. The right coronary (RC) and left coronary (LC) buttons are mobilized and the noncoronary (NC) sinus is excised. The aortic root is dissected and mobilized circumferentially as deep as possible. The membranous and muscular septum may limit the external dissection. The Valsalva graft (Vascutek Ltd, Terumo Aortic, Renfrewshire, UK) is then sized using the height of the LC–NC commissure. 11 The proximal aspect of the graft is trimmed corresponding to the anatomical limits of dissection at the base of the aortic root at the RC/LC and NC/RC commissures.

Twelve Ethicon 2–0 pledgeted sutures are passed underneath the annulus and passed through the proximal aspect of the graft following which the prosthesis is tied down. The aortoventricular junction is stabilized to 23 mm in men and 21 mm in women over a Hegar dilator. The commissures are reimplanted inside the graft 120-degree apart in patients with TAV and 180-degree apart in patients with BAV establishing a neo-STJ where the skirt of the Valsalva graft ends. A secondary continuous 4–0 polypropylene suture line is placed, securing the valve inside the graft. At this point, the valve is reassessed for prolapse by inspection and the use of the Schafers caliber and any residual prolapse is repaired with free edge plication. The coronary buttons are reattached and the distal anastomosis is constructed 1 cm proximal to the aortic cross-clamp (ACC), the heart is deaired, and the patient is taken off CPB.

In recent years, we have implemented several key adaptations to facilitate performing VSRR through an UHS. These include the use of Custodial–histidine–tryptophan–ketoglutarate and Del Nido cardioplegia, a modified cannulation strategy using the right femoral vein for venous inflow, and the use of Cor-Knot among other minimally invasive instruments. 14 16

### Follow-up

Patients were followed yearly after surgery with clinical visits either at our office or with their primary care physician or cardiologist. Echocardiography was performed yearly to assess valve function. Follow-up was obtained through a combination of institutional records, including outpatient clinical notes and echocardiograms, direct patient outreach, the use of the social security death index, and public obituary records.

### Statistical Analysis

Primary outcomes considered were all-cause mortality, aortic valve-related reoperation, and freedom from recurrence of AI. Kaplan–Meier estimates for freedom from mortality probability were constructed with study departure treated as a censoring event. A Kaplan–Meier estimate looking at a composite of reoperation, recurrence of AI, or death with study departure treated as a censoring event was constructed. Due to the low number of events in the UHS group, outcomes between the FS and UHS groups were not directly compared in formal hypothesis tests. Data were analyzed using R version 4.3.1.

## Results

### Patient Characteristics

The preoperative baseline characteristics are shown in Table 1 . A total of 81 patients undergoing VSRR using the reimplantation technique were included in this study. The patients were primarily male (89.3%) with mean age of 50.3 ± 1.4 years. Forty-nine patients had hypertension (60.5%), 6 had diabetes (7.4%), 8 had chronic obstructive pulmonary disease (9.9%), and 2 had prior stroke (2.5%). Six patients (7.4%) had previous cardiac operations and 2 patients (2.5%) had previous proximal aortic procedures. Four patients (4.9%) underwent redo sternotomy. Of the 10 patients (12.3%) who required VSRR for urgent or emergent indications, 8 (9.9%) had VSRR for acute type A aortic dissection and were performed exclusively through an FS.

**Table 1:** Preoperative baseline characteristics

Characteristics	Full ( *n* = 59)	Mini ( *n* = 22)	Total ( *n* = 81)
Patients ( *n* )	59	22	81
Age (y) (mean ± SE)	49.6 ± 1.7	52.1 ± 2.2	50.3 ± 1.4
Gender	53 (89.8%)	20 (90.9%)	73 (90.1%)
Race (Caucasian)	37 (62.7%)	18 (90%)	55 (69.6%)
Symptoms at presentation	33 (55.9%)	14 (63.6%)	47 (58%)
Pain	22 (37.3%)	3 (13.6%)	25 (30.9%)
Rupture	0 (0%)	0 (0%)	0 (0%)
Congestive heart failure	0 (0%)	0 (0%)	0 (0%)
NYHA dyspnea			
1 ( *n* , %)	2 (3.4%)	0 (0%)	2 (2.5%)
2	46 (78%)	19 (86.4%)	65 (80.2%)
3	6 (10.2%)	0 (0%)	6 (7.5%)
4	5 (8.5%)	2 (9.1%)	7 (8.6%)
NYHA angina			
1 ( *n* , %)	42 (77.8%)	21 (95.5%)	46 (78%)
2	6 (11.1%)	0 (0%)	6 (10.2%)
3	2 (3.7%)	1 (4.5%)	2 (3.4%)
4	2 (3.7%)	0 (0%)	3 (5.1%)
BMI (kg/m ^2^ )	28.3 ± 0.63	28.5 ± 1.1	28.37 ± 0.54
Associated disease			
Smoking	20 (33.9%)	8 (36.3%)	29 (34.5%)
Ascending aneurysm	58 (98.3%)	21 (95.5%)	79 (97.5%)
Acute dissection	8 (13.6%)	0 (0%)	8 (9.9%)
Chronic dissection	1 (1.7%)	1 (4.5%)	2 (2.5%)
Medial degeneration	36 (61%)	19 (86.4%)	55 (67.9%)
Marfan syndrome	7 (11.9%)	3 (13.6%)	10 (12.3%)
Bicuspid aortic valve	5 (8.5%)	3 (13.6%)	8 (9.9%)
Hypertension	34 (57.6%)	15 (68.2%)	49 (60.5%)
COPD	6 (10.2%)	2 (9.1%)	8 (9.9%)
Diabetes	5 (8.5%)	1 (4.5%)	6 (7.4%)
Cerebrovascular disease	4 (6.8%)	0 (0%)	4 (4.9%)
Prior stroke	2 (3.4%)	0 (0%)	2 (2.5%)
Prior transient ischemic attack	3 (5.1%)	0 (0%)	3 (3.7%)
Renal insufficiency	1 (1.7%)	0 (0%)	1 (1.2%)
Creatinine > 2.5	1 (1.7%)	0 (0%)	1 (1.2%)
Peripheral vascular disease	0 (0%)	1 (4.5%)	1 (1.2%)
Preoperative findings			
Root size (cm)	4.9 ± 0.09	5.2 ± 0.12	5.0 ± 0.07
Ascending size (cm)	4.4 ± 0.11	4.4 ± 0.13	4.4 ± 0.09
Annulus size (cm)	2.7 ± 0.09	2.7 ± 0.25	2.7 ± 0.08
Sinotubular junction size (cm)	4.1 ± 0.1	4.0 ± 0.29	4.0 ± 0.09
Arch size (cm)	3.2 ± 0.1	3.7 ± 0.18	3.3 ± 0.09
Urgent or emergent	9 (13.3%)	1 (4.5%)	10 (12.3%)
Previous proximal aortic surgery	2 (3.4%)	0 (0%)	2 (2.5%)
Previous distal aortic surgery	0 (0%)	0 (0%)	0 (0%)
Previous cardiac operations	4 (6.8%)	2 (9.1%)	6 (7.4%)
Ejection fraction	59 ± 1	58 ± 1.5	59 ± 0.8
Aortic insufficiency			
None ( *n* , %)	19 (32.2%)	12 (54.5%)	31 (38.3%)
Mild	20 (33.9%)	5 (22.7%)	25 (30.9%)
Moderate	13 (22%)	3 (13.6%)	16 (19.8%)
Severe	7 (11.9%)	2 (9.1%)	9 (11.1%)
Central (CM)	16 (27.1%)	3 (13.6%)	19 (23.5%)
Mitral regurgitation			
None ( *n* , %)	38 (64.4%)	15 (68.2%)	53 (65.4%)
Mild	16 (27.1%)	7 (31.8%)	23 (28.4%)
Moderate	4 (6.8%)	0 (0%)	4 (4.9%)
Severe	1 (1.7%)	0 (0%)	1 (1.2%)
Redo	3 (5.1%)	1 (4.5%)	4 (4.9%)

Abbreviations: BMI, body mass index; COPD, chronic obstructive pulmonary disease; NYHA, New York Heart Association; SE, standard error.

Most patients had a preoperative New York Heart Association Dyspnea class of II (80%). No AI, mild, moderate, and severe AI were present in 31 (38.3%), 25 (30.9%), 16 (19.8%), and 9 (11.1%) patients, respectively. Five patients (6.2%) had greater than mild mitral regurgitation. Mean aortic root size was 5.0 ± 0.07 cm and ascending aortic size was 4.4 ± 0.09 cm. Mean preoperative annulus size was 2.7 ± 0.08 cm and preoperative STJ size was 4.0 ± 0.09 cm. Eight patients (9.9%) had BAV and 10 patients (12.3%) had Marfan syndrome.

### Operative

Operative characteristics are displayed in Table 2 . Fifty-nine patients (72.8%) had VSRR performed through an FS and 22 patients (27.2%) had VSRR performed through an UHS. The most common Valsalva graft size was 32 mm (60.5%). Mean CPB time was 223 ± 5.0 minutes and mean ACC time was 193 ± 4.2 minutes.

**Table 2:** Operative characteristics

Characteristics	Full ( *n* = 59)	Mini ( *n* = 22)	Total ( *n* = 81)
Cardiopulmonary bypass time (min)	229.6 ± 6.1	204.9 ± 7.0	223.1 ± 5.0
Clamp time (min)	199.7 ± 5.0	174.5 ± 6.3	193.1 ± 4.2
Circulatory arrest time (min)	14 (10.5–20.5)	16.5 (8.75–24.25)	14 (10–21)
Incision type (full sternotomy)	59 (72.8%)	22 (27.2%)	81
Deep hypothermic circulatory arrest	18 (30.5%)	2 (9.1%)	20 (24.7%)
Blood loss (mL)	253 (0–555)	0 (0–89.5)	125 (0–500)
PRBCs (units)	0 (0–2)	0 (0–0)	0 (0–2)
Fresh frozen plasma (units)	2 (0–2.5)	0 (0–2)	0 (0–2)
Platelets (units)	1 (0–2)	1 (0–1.75)	1 (0–2)
Cryoprecipitate (units)	0 (0–2)	2 (0–4)	0 (0–4)
Simultaneous procedures	29 (49.2%)	19 (86.4%)	48 (59.3%)
CABG	1 (1.7%)	0 (0%)	1 (1.2%)
Mitral valve repair	3 (5.1%)	0 (0%)	3 (3.7%)
Patent foramen ovale closure	1 (1.7%)	0 (0%)	1 (1.2%)
Distal wrap	21 (35.6%)	15 (68.2%)	36 (44.4%)
Arch			
Hemiarch	5 (8.5%)	2 (9.1%)	7 (8.6%)
Trifurcation	4 (6.8%)	0 (0%)	4 (4.9%)
Elephant trunk + trifurcation	1 (1.7%)	0 (0%)	1 (1.2%)
Annulus postoperative (cm)	2.2 ± 0.02	2.2 ± 0.14	2.2 ± 0.03
Sinotubular junction postoperative size (cm)	3.0 ± 0.07	2.9 ± 0.13	3.0 ± 0.06

Abbreviations: CABG, coronary artery bypass grafting; PRBC, packed red blood cell.

Right coronary cusp (RCC) prolapse was most common ( *n* = 17, 21.0%), followed by Non-coronary cusp (NCC) prolapse ( *n* = 11, 13.6%), and left coronary cusp (LCC) prolapse ( *n* = 7, 8.6%). There were 24 (29.6%) patients who had leaflet repair. Leaflet repair characteristics are displayed in Table 3 . RCC, LCC, and NCC plication were performed in 21 (25.9%), 13 (16%), and 4 (4.9%) patients, respectively. There were 11 (13.6%) patients, 11 (13.6%) patients, and 2 (2.5%) patients that had 1, 2, and 3 leaflet plication respectively. Subcommissural annuloplasty between the NCC and LCC was performed in 1 (1.2%) patient. The LCC was the most common location of fenestrations ( *n* = 3, 3.7%). Twelve (14.8%) patients had residual prolapse after reimplantation that required additional leaflet plication after evaluation of the leaflets with the Schafers caliper. Immediately postrepair, there were no cases of moderate or severe central AI or eccentric AI, with only 10 patients (12.3%) having trivial to mild central AI. Mean leaflet coaptation height was 10.3 ± 0.5 mm.

**Table 3:** Valve repair techniques

Characteristics	Full ( *n* = 59)	Mini ( *n* = 22)	Total ( *n* = 81)
Coaptation height (mm)	10.0 ± 0.5	12.0 ± 0.9	10.3 ± 0.5
Graft size			
30	17 (30.9%)	4 (19%)	21 (27.6%)
32	32 (58.2%)	14 (66.7%)	46 (60.5%)
34	3 (5.5%)	2 (9.5%)	5 (6.6%)
RCC prolapse	12 (20.3%)	5 (22.7%)	17 (21.0%)
RCC fenestration	1 (1.7%)	0 (0%)	1 (1.2%)
RCC plication	16 (27.1%)	5 (22.7%)	21 (25.9%)
RCC edge suspension	2 (2.4%)	1 (4.5%)	3 (3.7%)
LCC prolapse	6 (10.2%)	1 (4.5%)	7 (8.6%)
LCC fenestration	3 (5.1%)	0 (0%)	3 (3.7%)
LCC plication	11 (18.6%)	2 (9.1%)	13 (16%)
LCC edge suspension	2 (3.4%)	0 (0%)	2 (2.5%)
NCC prolapse	7 (11.9%)	4 (18.2%)	11 (13.6%)
NCC fenestration	0 (0%)	0 (0%)	0 (0%)
NCC plication	0 (0%)	1 (4.5%)	4 (4.9%)
NCC edge suspension	0 (0%)	0 (0%)	0 (0%)
1 leaflet prolapse	8 (13.6%)	3 (13.6%)	11 (13.6%)
2 leaflet prolapse	4 (6.8%)	2 (9.1%)	6 (7.4%)
3 leaflet prolapse	3 (5.1%)	1 (4.5%)	4 (4.9%)
1 leaflet plication	9 (15.3%)	2 (9.1%)	11 (13.6%)
2 leaflet plication	8 (13.6%)	3 (13.6%)	11 (13.6%)
3 leaflet plication	2 (2.5%)	0 (0%)	2 (2.5%)
NC-LC annuloplasty	0 (0%)	1 (4.5%)	1 (1.2%)
LC-RC annuloplasty	0 (0%)	0 (0%)	0 (0%)
RC-NC annuloplasty	0 (0%)	0 (0%)	0 (0%)
Correction of residual prolapse	11 (18.6%)	1 (4.5%)	12 (14.8%)

Abbreviations: LC, left coronary; NC, noncoronary; RC, right coronary.

Major concomitant procedures include coronary artery bypass grafting ( *n* = 1, 1.2%), mitral valve surgery ( *n* = 3, 3.7%), and arch surgery ( *n* = 12, 14.8%). Overall usage of blood products, including packed red blood cells (0 [interquartile range, IQR: 0–2]), fresh frozen plasma (0 [IQR: 0–2]), platelets (1 [IQR: 0–2]), and cryoprecipitate (0 [IQR: 0–4]) was minimal.

### Early Outcomes

There was no 30-day or in-hospital mortality ( Table 4 ). Additionally, median intensive care unit stay was 3 (2–5) days and median hospital stay was 7 (6–9) days. One (1.2%) patient had a postoperative transient ischemic attack and 1 patient (1.2%) had a stroke. Ten patients (12.3%) required prolonged ventilation support (>48 hours), 7 patients (8.6%) developed atrial fibrillation, and 6 patients (7.4%) required reoperation for bleeding. One patient (1.2%) developed new renal insufficiency.

**Table 4:** Early postoperative outcomes

Characteristics	Full ( *n* = 59)	Mini ( *n* = 22)	Total ( *n* = 81)
ICU stay (d)	4 (2–5)	3 (2–4)	3 (2–5)
Hospital stay (d)	7 (6–11)	6 (6–7)	7 (6–9)
Prolonged ventilatory support	8 (13.6%)	2 (9.1%)	10 (12.3%)
Chest left open	2 (3.4%)	1 (4.5%)	3 (3.7%)
Stroke	1 (1.7%)	0 (0%)	1 (1.2%)
Transient ischemic attack	1 (1.7%)	0 (0%)	1 (1.2%)
New renal insufficiency	1 (1.7%)	0 (0%)	1 (1.2%)
Postoperative bleeding	5 (8.5%)	1 (4.5%)	6 (7.4%)
Vocal cord paralysis	2 (3.4%)	0 (0%)	2 (2.5%)
Temporary neurological dysfunction	2 (3.4%)	0 (0%)	2 (2.5%)
Myocardial infarction	0 (0%)	0 (0%)	0 (0%)
Congestive heart failure	0 (0%)	0 (0%)	0 (0%)
Arrhythmia	7 (11.9%)	0 (0%)	7 (8.6%)
Pacemaker	0 (0%)	1 (4.5%)	1 (1.2%)
Effusion	6 (10.2%)	1 (4.5%)	7 (8.6%)
Atelectasis	1 (1.7%)	0 (0%)	1 (1.2%)
Tracheostomy	1 (1.7%)	0 (0%)	1 (1.2%)
Reintubation	2 (3.4%)	0 (0%)	2 (2.5%)
Mortality	0 (0%)	0 (0%)	0 (0%)
Postoperative aortic insufficiency			
None	52 (88.1%)	19 (86.4%)	71 (87.7%)
Mild	7 (11.9%)	3 (13.6%)	10 (12.3%)
Moderate	0 (0%)	0 (0%)	0 (0%)
Severe	0 (0%)	0 (0%)	0 (0%)

### Late Outcomes

**Fig. 1:**
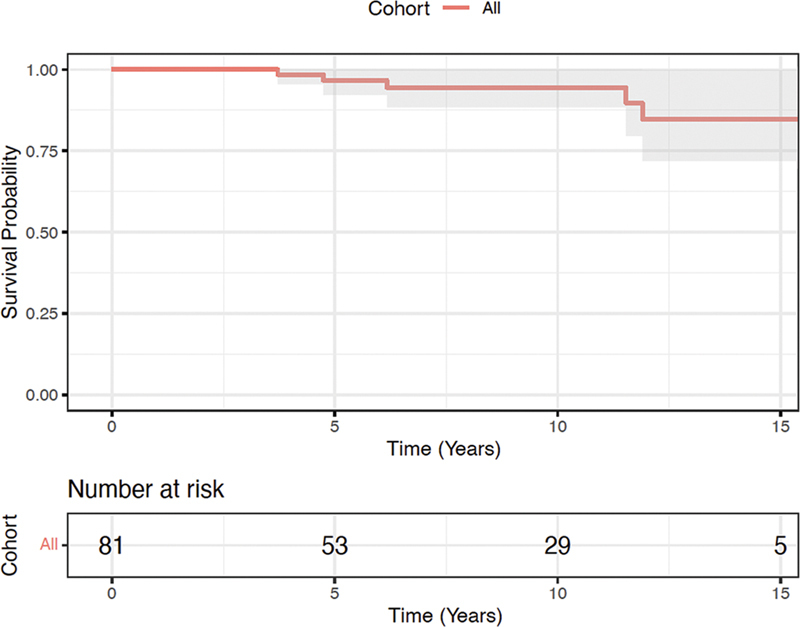
Freedom from mortality.

Late outcomes are displayed in Table 5 . The mean follow-up length was 7.6 ± 0.5 years with 29 (35.8%) patients being followed for >10 years and 5 (6.2%) patients being followed for >15 years. Clinical follow-up was complete and echocardiographic follow-up was 89% complete. On follow-up echocardiography, average mean and peak gradient were 5 (3–8) and 10 (6–16) mm Hg, respectively. Freedom from all-cause mortality at 1, 5, and 10 years was 100, 96.6 and 94.4%, respectively ( Fig. 1 ). There were five late deaths, of which four were confirmed not to be cardiac-related (coronavirus disease, meningioma, stroke, unspecified neurological condition). Cause of death could not be determined for one patient.

**Table 5:** Mid-term outcomes

Characteristics	Full ( *n* = 59)	Mini ( *n* = 22)	Total ( *n* = 81)
Follow-up length (y)	8.7 ± 0.64	4.6 ± 0.4	7.6 ± 0.5
Freedom from mortality			
1 y	100%	100%	100%
5 y	97.70%	94.10%	96.60%
10 y	95.20%		94.40%
Freedom from recurrence of aortic insufficiency			
1 y			100%
5 y			100%
10 y			97.3%
Freedom from reoperation			
1 y			98.8%
5 y			95.4%
10 y			95.4%
Ejection fraction long-term	60.32 ± 1.22	58.4 ± 2.0	59.72 ± 1.1
LVEDD (cm)	5.1 ± 0.14	4.26 ± 0.23	4.91 ± 0.14
LVESD (cm)	3.41 ± 0.13	2.89 ± 0.19	3.3 ± 0.11
Aortic root size (cm)	3.64 ± 0.08	3.92 ± 0.12	3.75 ± 0.06
Mean gradient (mm Hg)	5 (3.1–7)	7.5 (4.5–10.25)	5.3 (3.1–8)
Peak gradient (mm Hg)	10 (6.4–16)	10 (6–15)	10 (6.1–15.75)

**Fig. 2:**
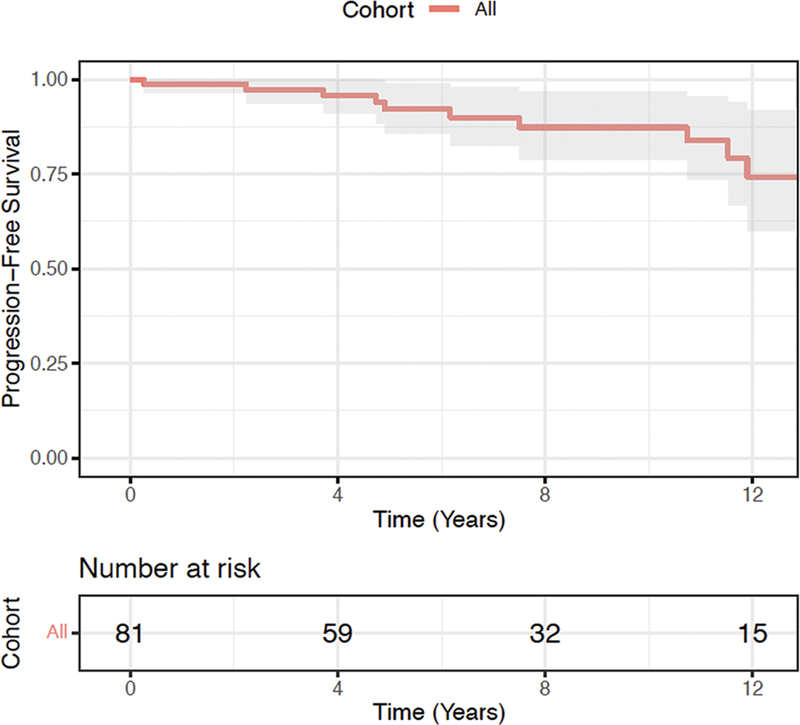
Composite freedom from mortality, aortic valve-related reoperation, and recurrence of AI.

Cumulative freedom from mortality, freedom from reoperation, and freedom from recurrence was 98.8, 92.1, and 87.3% at 1, 5, and 10 years ( Fig. 2 ). Four patients required aortic valve-related late reoperation at 0.25, 2.2, 4.9, and 16.0 years. There were no late reoperations for recurrence of greater than mild AI. The first patient developed an aortic root abscess a few months following surgery ultimately requiring a Bentall procedure with a bioprosthetic valve. The second patient developed endocarditis and ultimately received a transcatheter aortic valve replacement (TAVR). The third patient developed ventricular tachyarrhythmia following surgery and had an iatrogenic aortic leaflet perforation following a transcatheter intervention requiring an aortic valve replacement. The final patient developed severe aortic stenosis and received a TAVR. Four patients developed late recurrence of new mild-to-moderate central AI at 7.5, 13.0, 13.1, and 16.9 years. All four patients were asymptomatic. There was no recurrence of moderate or severe AI in the follow-up period.

## Discussion

### Outcomes

Since the first series of VSRR described by David and colleagues, multiple series, including David's own 25-year experience, have demonstrated excellent outcomes, with <1% operative mortality and 10-year valve-related freedom from reintervention > 90%. 17 18 19 20 21 Our results are consistent with the literature with no operative mortality and an overall survival of 94.4% at 10 years, which is excellent considering the inclusion of patients with aortic dissection and redo procedures. Moreover, none of the deaths were confirmed to be cardiac-related. One (1.2%) patient who underwent VSRR for acute type A aortic dissection developed a stroke and had near resolution of symptoms by postoperative day 8 and no patients developed serious cardiac complications. The low perioperative morbidity can be partially attributed to the fact that we are very selective in performing this operation primarily in younger individuals with low comorbid conditions and adhere to strict technical details to prevent perioperative bleeding or coronary malperfusion. In addition, we maintain tight blood pressure control (systolic blood pressure < 120 mm Hg and diastolic blood pressure < 80 mm Hg) during the hospital course.

Due to the limited size of the series, a composite of mortality, reoperation, and recurrence of AI was calculated in lieu of individual analysis of reoperation and recurrence of AI and found to be 87.3% at 10 years, which compares favorably to the established >90% freedom from reoperation at 10 years surgical quality standards set by the National Marfan Foundation. David and colleagues have reported freedom from AI of 93.2 and 78% at 10 and 18 years and freedom from aortic valve reoperation of 97.0 and 94.8% at 10 and 18 years. 19 The largest series of VSRR procedures reported an 8.2% recurrence of AI across the follow-up period, with 85% of those patients subsequently necessitating reintervention at 15 years. 20 Notably, only 4 (4.9%) of our patients developed recurrence of mild-to-moderate AI during the follow-up period with no recurrence of moderate AI or greater. Three out of the four recurrences occurred after 10 years, and there were no late reinterventions for recurrence of AI. Of the four late reinterventions, the aortic valve replacement for iatrogenic leaflet perforation from a transcatheter procedure was potentially avoidable.

In our opinion, our cohort has had excellent valve durability for several reasons. First, we are highly selective with the patients we choose to perform VSRR in. We do not perform VSRR in patients with any calcification in TAV valves or more than mild raphe calcification in bicuspid valves, or any aortic stenosis. Also, patients with large fenestrations particularly associated with prolapse are excluded. Small fenestrations are not a contraindication. To address leaflet prolapse, we perform mostly central free-edge plication and rarely leaflet resuspension. In patients with pliable leaflets with a thicker free edge, we perform leaflet shaving with an 11-blade followed by central free edge plication if needed. In patients requiring extensive patch repair, which is associated with early repair failure, we will elect to replace the valve. 22 23 On preoperative echocardiographic evaluation, 21 (25.9%) patients had 1 or more prolapsed leaflets. Intraoperatively, 24 (29.6%) patients required leaflet plication, with 11 (13.6%) patients, 11 (13.6%) patients, and 2 (2.5%) patients needing 1, 2, and 3 leaflet plication, respectively. Twelve (14.8%) patients required additional plication for residual prolapse following valve reimplantation. One (1.2%) patient was placed back on bypass before leaving the operating room for additional leaflet plication after weaning because of the development of moderate AI. Nearly half of our patients that required leaflet repair required additional leaflet repair after reimplantation. These results suggest that valves need to be carefully inspected for residual prolapse even after reimplantation as there can be induced prolapse. Without aggressively addressing leaflet prolapse both before and after reimplantation, patients may be more prone to developing early repair failure and recurrence of AI. Thus, we never leave the operating room with greater than mild central AI.

### Modifications and Reproducibility

Over the years, various modifications have been made to not only better mimic the physiology of the native aortic root, but also to increase reproducibility. The original “David I” utilized a straight tube graft, which increased aortic cusp closing velocities causing increased stress and theoretically, quicker degeneration of the leaflets. The current “David V” procedures utilizes a larger and smaller graft create a “pseudosinus,” which has more physiological leaflet mechanics. 24 25

We believe that the “El Khoury” technique, which utilizes Valsalva grafts, allows for the most reproducible procedure without sacrificing outcomes. We always remove the collar of the Valsalva graft. The transition line between the skirt and the body of the Valsalva graft effectively serves as a well-demarcated neo-STJ, which allows for reproducible reimplantation of the commissures every time. To account for anatomical differences in the height of the commissures, the proximal aspect of the skirt is trimmed in the areas of the LC/NC and NC/RC commissures, if needed to respect the natural anatomical limits of dissection of the aortic root. There are also markings at 120 degrees, which allow for the most consistent commissure positioning at reimplantation. Although the David V and Stanford medication allows the surgeon to better customize the pseudosinuses to each patient, improved long-term durability has not been demonstrated compared with the use of commercially available Valsalva grafts. Routine utilization of Schafers caliper to look for an effective height > 8 mm has also resulted in a more systematic, reproducible assessment for prolapse compared with visual inspection.

### Minimally Invasive Experience

Since 2015, we have adopted an UHS first approach for all patients undergoing elective or urgent VSRR. In the follow-up period, in our 22 patients, there were no cardiac-related deaths and one reoperation at 3 months due to a mediastinal abscess. Monsefi et al, in 26 UHS patients with mean follow-up time of 3 ± 2 years, reported no operative mortalities and 1 reintervention for recurrence of severe AI in the follow-up period. 12 Similarly, Shrestha et al, in 42 UHS patients with mean follow-up time of 4.2 ± 2.1 years, reported an operative mortality of 2.4% with 1 late mortality and 3 late reinterventions. 13 Our results are consistent with the previous conclusions, that in the midterm, VSRR through an UHS is an acceptable operation, although long-term durability is still to be determined.

## Limitations

This was a retrospective review of a large prospectively maintained aortic registry and is limited by smaller sample size. With the low number of observed events, direct comparison was also not made directly between FS and UHS groups due to limited statistical power. Given this is a single-surgeon series, selection bias may be present and results may not be generalizable. Also, we were unable to obtain the latest echocardiographic data on a small group of patients, although at the latest clinical follow-up these patients were asymptomatic. In addition, longer follow-up is still needed to determine long-term valve durability in patients undergoing VSRR through an UHS.

## Conclusion

VSRR using the reimplantation technique is a durable procedure that when performed properly and judiciously, carries a low mid-term mortality. It can be performed safely in high-risk patients, including those with acute type A aortic dissection, BAV, Marfan syndrome, or requiring redo operation. Efforts to standardize aspects of the procedure, including graft sizing, commissure placement, and assessment of prolapse have increased the reproducibility of the procedure. Although minimally invasive approaches are promising, long-term follow-up is necessary to determine durability.

## Central Message

This study examined 81 patients undergoing David procedure using the reimplantation technique for diverse indications. With careful preoperative selection, VSRR is a durable procedure for patients with aortic root aneurysm.
